# Obese Locus in WNIN/Obese Rat Maps on Chromosome 5 Upstream of Leptin Receptor

**DOI:** 10.1371/journal.pone.0077679

**Published:** 2013-10-21

**Authors:** Rajender Rao Kalashikam, Kiran Kumar Battula, Veerababu Kirlampalli, Jeffrey M. Friedman, Giridharan Nappanveettil

**Affiliations:** 1 Molecular Genetics, National Centre for Laboratory Animal Science (NCLAS), National Institute of Nutrition (NIN), Hyderabad, India; 2 Molecular Genetics Laboratory, Howard Hughes Medical Institute, Rockefeller University, New York, New York, United States of America; CSIR-Central Drug Research Institute, India

## Abstract

WNIN/Obese (WNIN/Ob) rat a new mutant model of metabolic syndrome was identified in 1996 from an inbred Wistar rat strain, WNIN. So far several papers are published on this model highlighting its physical, biochemical and metabolic traits. WNIN/Ob is leptin resistant with unaltered leptin or its receptor coding sequences - the two well-known candidate genes for obesity. Genotyping analysis of F_2_ progeny (raised from WNIN/Ob × Fisher - 344) in the present study localized the mutation to a recombinant region of 14.15cM on chromosome 5. This was further corroborated by QTL analysis for body weight, which narrowed this region to 4.43 cM with flanking markers D5Rat256 & D5Wox37. Interval mapping of body weight QTL shows that the LOD score peak maps upstream of leptin receptor and shows an additive effect suggesting this as a novel mutation and signifying the model as a valuable resource for studies on obesity and metabolic syndrome.

## Introduction

Obesity, referred to as overweight due to excess fat accumulation has assumed epidemiological proportion in the last decade [1,2] along with its associated disorders [3-6]. Medical world is now grappling with this modern malady and strategies are being evolved to tackle this problem. Development of several animal models over the years in different parts of the world has advanced our knowledge on obesity [7-14], yet the quest to overcome the syndrome X has been elusive demanding for novel natural mutant models. 

National Centre for Laboratory Animal Science (NCLAS) at National Institute of Nutrition (NIN), India is maintaining one of the oldest Wistar rat stocks (since 1920), designated as WNIN. During 1990’s, a ‘Fat’ rat with about 47% of its body weight representing fat was identified from this stock, and later by pedigree and back cross analysis, a mutant strain was established as a uni-locus model for obesity trait, designated as WNIN/Ob. The pattern of inheritance was found to be autosomal incomplete dominance and the strain exhibited three phenotypes viz., lean, carrier (heterozygote) and obese, inherited in a Mendelian fashion, identifiable by morphology, body weight as well as by a kinky tail association (only in carriers and homozygous obese). Incomplete dominance and co-segregation of kinky tail are unique characteristics shown by this model in comparison to the existing rodent models of obesity [15]. It is the biggest rat (1.4Kg) of its genre ever recorded in the world [16,17]. With features like hyperphagia, hypertriglyceridemia, hypercholesterolemia and hyperleptinemia, it turned out to be a worthwhile model for metabolic syndrome, with accelerated ageing and degenerative disorders like impaired immunity, tumours, infertility, polycystic ovaries, cataract and retinal degeneration [18-21]. Coding DNA sequences of the known genes of obesity like leptin and leptin receptor seems to be unaltered in this animal model [22]. Our preliminary genome scan performed in the F2 progeny of a cross between WNIN/Ob x Fisher 344, using microsatellite markers, spanning an average genetic distance of 20cM interval , led to the identification of a marker- D5Wox256 - on chromosome No.5 showing association with obese trait. The present analysis reported here is our attempt to fine map this region further with an objective to narrow down the obese locus in question.

## Materials and Methods

### Animal Experiments

#### Ethical consent

Animal experiments in the study were conducted after obtaining consent from Institutional Animal Ethical committee (IEAC), NCLAS, NIN.

#### Genetic Resource

Rat strains employed for the study were bred and maintained at NCLAS, NIN. Food restricted male obese rats (competent for reproduction) from WNIN/Ob (n= 5) and female rats from Fischer-344(n=13) representing F_0_ generation were crossed to raise F_1_ progeny. F_1_ progeny rats (20 males x 40 females) were inter-crossed to raise F_2_ progeny.

#### Maintenance of Rats

Rats were housed in clean polypropylene cages with sterilized paddy husk as bedding material. Room temperature was maintained at 22±2°C with 14-16 air changes per hour and 55±5% relative humidity. Rats were maintained with 12 hour light-dark cycles and the animals had free access to food and water.

### Analysis

#### Test for Mendelian inheritance

Chi-square (χ^2^) test was conducted in F_2_ progeny to test for the expected 1:2:1 ratio of lean, carrier and obese phenotypes respectively. Observed ratios were considered significant for χ^2^ value < 5.99 (df=2; p-value > 0.05). Phenotypes were identified based on morphological characteristics, body weights and kinky tail association.

####  Body weight measurements and statistics

Body weights of the rats were recorded at 3 months age using standard electronic balance with a precision of 1gm (Sartorius, Germany). Descriptive statistics like range, inter-quartile range and Median values were used to describe body weights of the observed phenotypes. Kruskal-Wallis (χ^2^) and Wilcoxon rank sum tests (W) were performed to infer body weight differences. Test statistics with p-value ≤ 0.05 were considered significant. Analyses were carried out on ‘R’ software through ‘R Commander’ [23].

#### Genomic DNA isolation

Tails were clipped from F_0_ and F_2_ rats and genomic DNA was isolated from them using Qiagen genomic DNA isolation kit.

#### Genetic Markers

Five microsatellite DNA markers polymorphic between WNIN/Ob and Fischer-344(F-344) spanning genetic distance of 31cM (as per SHRSPXBN genetic map, Rat Genome Database [24]) and flanking D5Rat256 were selected for the study. Upstream flanks were D5Rat98, D5Got131 and downstream flanks were D5Wox37, D5Rat235, and D5Rat69. Primer sequences for the above markers were obtained from Rat Genome Database [25].

#### Genotyping

Genotyping was performed on ABI-377 machine at Rockefeller University. A total of 68 F_2_ progeny (66 obese+2carriers) were genotyped. Genotypes were constructed by scoring for alleles of WNIN/Ob and F-344. Rats showing alleles of WNIN/Ob were coded as ‘A’, Rats showing alleles of F-344 were coded as ‘B’ and Rats showing alleles of both WNIN/Ob and F-344 were coded as ‘H’.

#### Tests for association of markers with obese trait

The genotyped F_2_ obese rats at each marker comprised about 63-66 (126 to 132 meioses). Recombination fractions (θ) were calculated as: number of meiotic recombinations detected / number of meioses scored. χ^2^ tests were conducted for each marker to determine deviation of the observed number of meiotic recombinations (1*H, 2*B) from the expected 50% . χ^2^ value > 10.83 (df=1; p<0.001) were considered significant. Logarithm of Odds (LOD) scores were calculated to determine linkage using the formula, Z = (((1-θ)^NR)*( θ^R))/(0.5^NR+R), where NR is the number of non-recombinants and R is the number of recombinants [26]. F_2_ obese rats showing ‘A’ genotype at a marker were scored as non-recombinants and ‘H’ or ‘B’ genotype as recombinants. LOD scores ≥ 3 were considered significant. As LOD scores cannot be calculated for ‘θ’ = 0, the value was set to 0.001 for the respective marker assuming less than 0.01% recombination fraction. Estimates of linkage disequilibrium (Dꞌ) and its correlation coefficient (r^2^) were obtained through ‘Gentics’ package for ‘R’ software [27]. As calculations could not be performed for marker loci with single allele, a rare allele was introduced to one of the individuals at the respective marker loci. Dꞌ > 0.6 suggest for linkage disequilibrium. r^2^ = 1 suggest for perfect linkage disequilibrium. 

#### Estimation of Genetic map distances

ONEMAP package for ‘R’software [28] was used for the analysis. F_2_ inter-cross data was prepared as per format and piped for the following estimations. Recombination fractions were estimated between all pairs of markers using two point tests with threshold LOD score of 4 and maximum recombination fraction of 3.5. Markers were assigned to linkage groups using the same above thresholds. Kosambi mapping function was used to display the genetic map. Markers of the linkage group were ordered by two-point based algorithm (Seriation). A framework of ordered markers was derived by comparing all possible orders using ‘Compare’ function. ‘Ripple’ function was used to permute sequentially with 4 markers per subset to check for any plausible alternative orders.

### QTL analysis

#### Test for Association of marker with Phenotype (bodyweight)

To determine association between the marker and bodyweight phenotype, single factor analysis of variance was conducted on body weights of F_2_ progeny rats with ‘A’ genotype versus ‘H’ genotype. Microsoft Excel-2007 was used for the above analysis. Association was considered significant for p< 0.001. 

#### Interval mapping

R/QTL package was used for the analysis [29]. Markers with genotype, phenotype, and map distance data (estimated using ONEMAP package) were imported into R/QTL. Genotype probabilities were calculated every 0.2cM interval to represent the interval map. QTL mapping was carried under normal model using EM algorithm. One thousand permutations were conducted to determine LOD significance thresholds and p-value for the peak LOD score. Confidence intervals for the QTL were generated by conducting 1000 bootstrap replicates. Additive and dominant effects of the QTL were estimated. Proportion of phenotypic variance (R^2^) for the linked markers was determined using QTL cartographer [30]. 

## Results and Discussion

**Figure 1:**
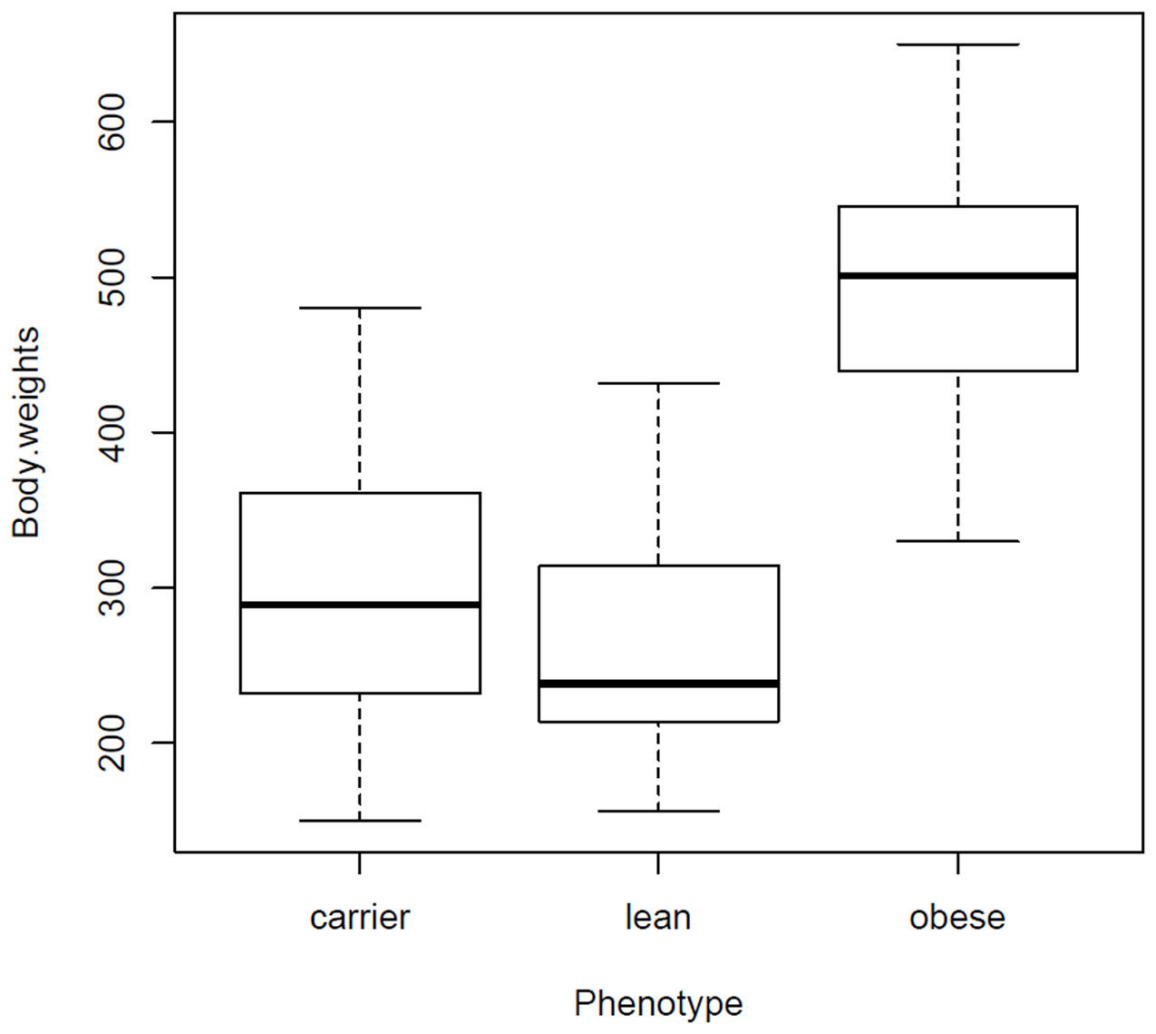
Boxplot showing distribution of body weights in the three phenotypes of F_2_ progeny rats, Lean(n=77), Carrier(n=151) and Obese(n=71).

The mutation reported in WNIN/Ob is uni-locus with three differentiating phenotypes, i.e., lean^(+/+)^, carrier^(+/-)^ and obese^(-/-)^ [15]. The F_2_ progeny in the present study represented a total of 305 rats among which lean were 77, carriers were 151 and obese were 77. Test for Mendelian inheritance of obese trait suggested that F_2_ progeny follows 1:2:1 ratio (χ^2^=0.052). The probability of occurrence of Mendelian ratio calculated from the present study is more than 95% (p>0.95; df=2). The mutation causing obesity in WNIN/Ob was reported as incomplete dominant where, rats of carrier phenotype were comparatively heavier than their lean counterparts [15]. F_2_ progeny in the present analysis showed a similar trend. Body weights (gms) of F_2_ progeny ranged from 156 - 432 in lean rats, 150 - 480 in carrier rats, and 330 - 650 in obese rats. The likely range of variation i.e., Inter Quartile Range was 214 - 314 in lean, 232 - 361 in carriers and 440 - 546 in obese rats. Median values were 238, 289, and 501 for lean, carrier and obese rats respectively (Figure 1). Kruskal-Wallis test suggested a significant difference (χ^2^=165.134, df = 2, p-value < 2.2e-16) between the phenotypes. Pairwise comparison by Wilcoxon Rank Sum test showed significant differences for lean versus obese (W=46.5, p-value<2.2E-16), lean versus carrier (W=7485, p-value=4.8E-04) and carrier versus obese (W=331.5, p-value<2.2E-16). These results re-confirm or previous findings on Mendelian inheritance and incomplete dominant nature of obese mutation identified in WNIN/Ob. 

Preliminary analysis on genome wide scan in F_2_ progeny, identified a genetic marker (D5Rat256) associated with obese phenotype. With the objective to narrow down towards mutation causing obesity, five additional markers were genotyped in the vicinity in F_2_ progeny. All the six markers, D5Rat98, D5Got131, D5Rat256, D5Wox37, D5Rat235, D5Rat69 showed less than 50% recombination with obese locus as indicated by their recombination fractions (θ) suggesting for an association. Chi-square test conducted for the above, showed a significant association for five markers D5Rat98, D5Got131, D5Rat256, D5Wox37, and D5Rat235. Linkage analysis revealed a significant linkage of the markers, D5Got131, D5Rat256, D5Wox37, and D5Rat235 with obese locus as indicated by their LOD scores. Highest LOD score was observed for D5Wox37 suggesting for a tight linkage. All the markers showed linkage disequilibrium with obese locus where as only one marker, D5Wox37 showed perfect disequilibrium as explained by the r^2^ value (Table 1)

**Table 1:** Association test scores for obese trait vs markers.

Paired loci	θ	χ^2^	LOD score	D'	r^2^
Ob-D5Rat98	0.13	14.06*	1.36	0.94*	-0.03
Ob-D5Got131	0.08	28.45*	5.55*	0.91*	-0.02
Ob-D5Rat256	0.05	42.25*	10.17*	0.83*	-0.02
Ob-D5Wox37	0.001	66.00*	19.84*	0.99*	0.99*
Ob-D5Rat235	0.06	36.94*	8.26*	0.87*	-0.02
Ob-D5Rat69	0.21	1.29	-3.02	0.96*	-0.04

Recombination fraction (θ) is the number of meiotic recombinations detected/number of meioses scored. χ^2^ value with * suggest that the observed number of meiotic recombinations differ significantly from 50% with p<0.001. LOD score > 3 suggest for a significant linkage. D' values with * suggest that the paired loci showed linkage disequilibrium. r^2^ value with * explains the perfect disequilibrium.

**Figure 2:**
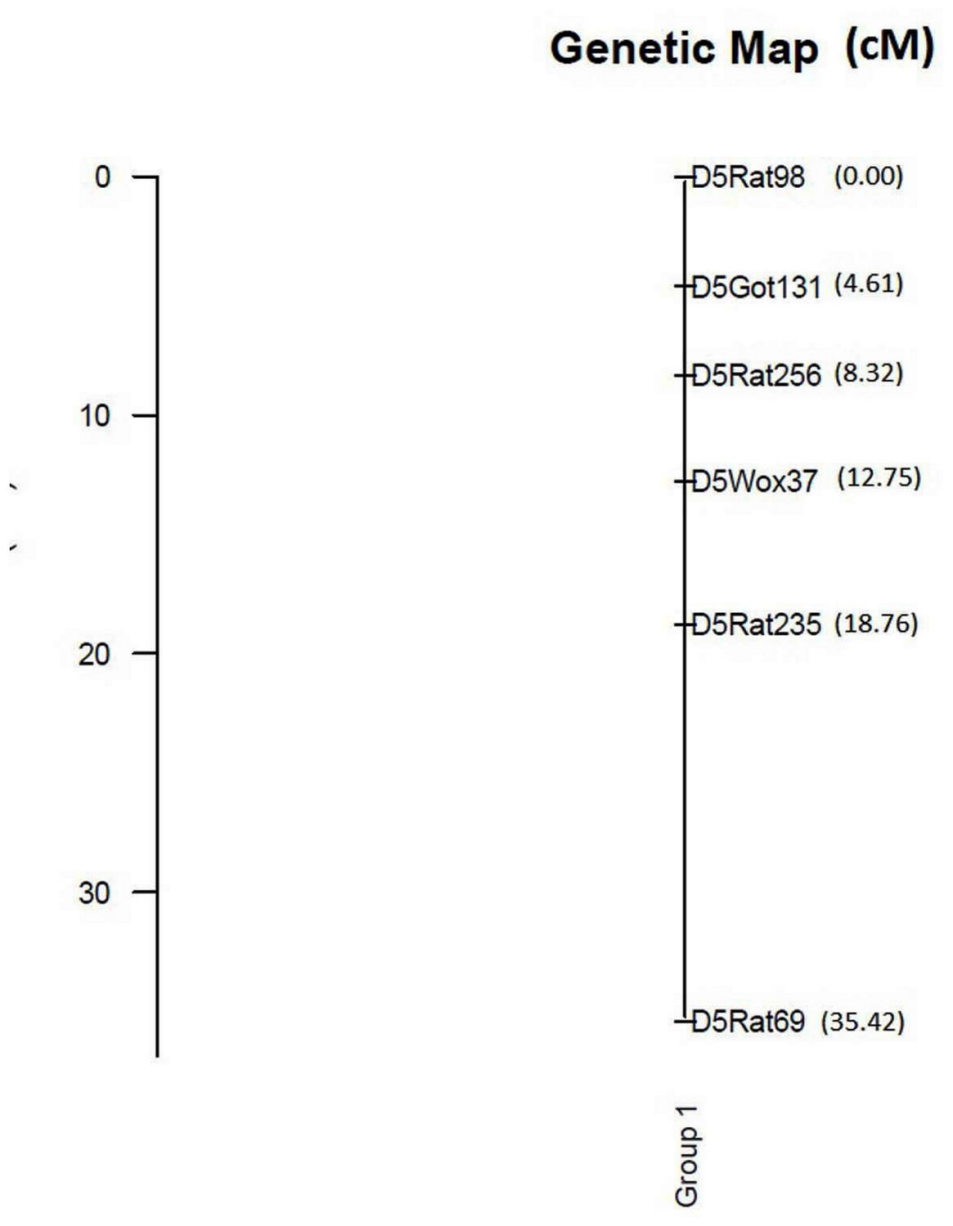
Genetic map distances (cM) were estimated between the markers using F_2_ progeny genotype data (n=68).

To estimate genetic map distances between the six markers, the genotype data of F_2_ progeny (Table S1) was subjected to linkage analysis. Recombination fractions estimated between all the marker pairs were less than 0.24. Assignment of markers to linkage groups resulted in formation of a single linkage group. Ordering of markers resulted in the following sequence: D5Rat98 - D5Got131 - D5Rat256 - D5Wox37 - D5Rat235 - D5Rat69. Log-likelihood ratio observed for the above sequence was -217.5473. No plausible alternative order of markers was detected from permutation tests. The order of markers obtained from the above analysis is in agreement with their physical map positions [31]. Genetic distance of 18.76cM was observed between the markers D5Rat98 and D5Rat235 in the present analysis, against known distance of 14.5cM (SHRSP X BN cross [24]) and a genetic distance of 16.66cM was observed between D5Rat235 and D5Rat69, against known distance of 17cM (SHRSP X BN cross [24]). The increase in genetic distance between D5Rat98 and D5Rat235 observed in the present analysis could be mainly due to increase in the markers density. This study reports for the first time the genetic distances for the markers D5Got131, D5Rat256, D5Wox37 with known mapped markers, D5Rat98, D5Rat235, D5Rat69 (Figure 2). The markers showing significant linkage with obese locus spanned a genetic distance of 14.15cM (D5Got131- D5Rat235).

**Figure 3:**
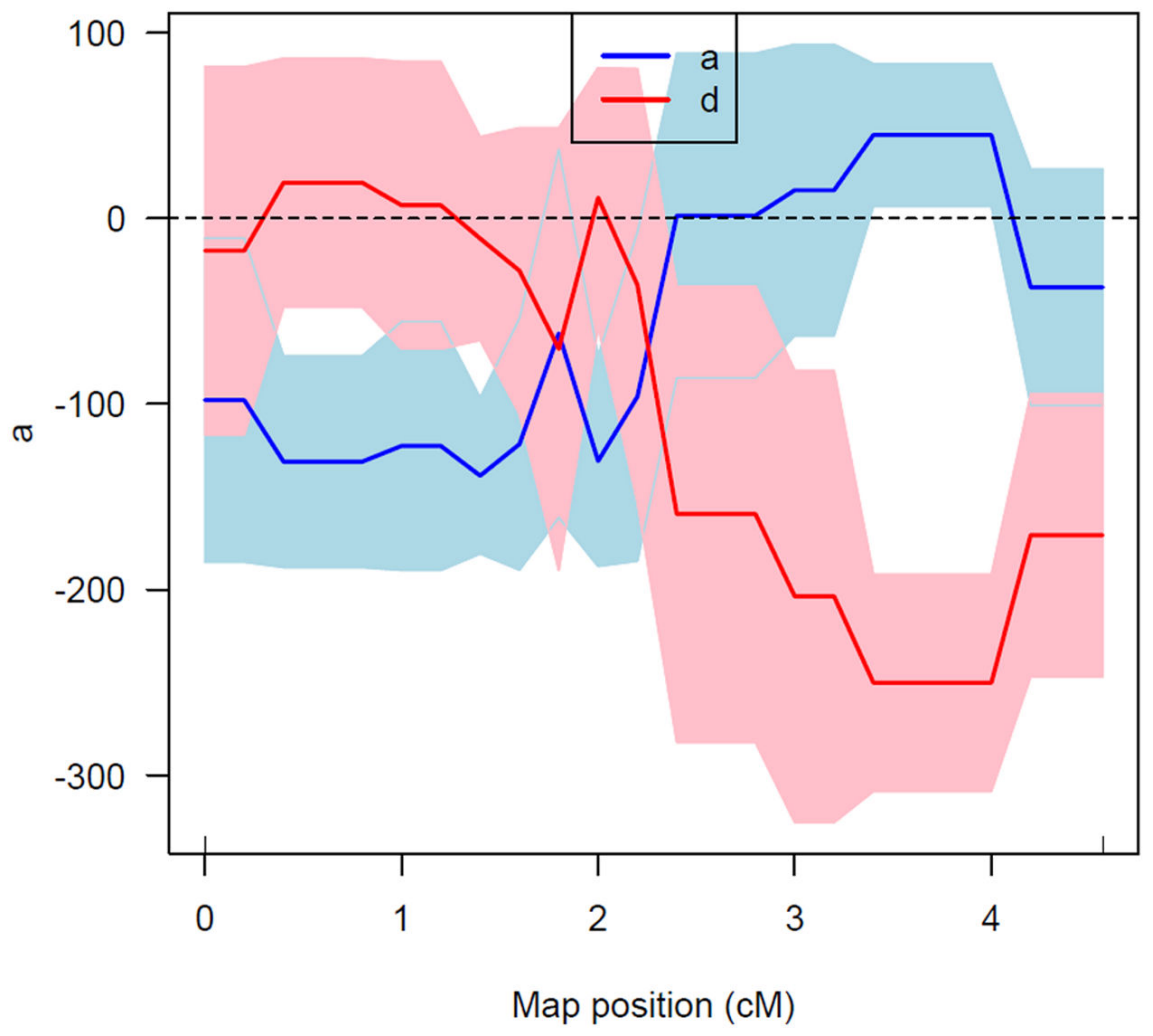
Effect scan at 0.2cM interval between the markers D5Rat256 and D5Wox37 explains the additive effect of the putative QTL.

To further analyze the genomic region that is associated with obese trait, quantitative trait locus (QTL) mapping of the incomplete dominant phenotype (body weight) was carried out. As discussed earlier, rats with ‘Obese’ phenotype showed significantly higher body weights compared to their ‘Carrier’ or ‘Lean’ counter parts and ‘Carriers’ were comparatively heavier than their ‘Lean’ counterparts. To determine whether a quantitative trait locus for the excess body weight exist in the currently analyzed genomic region, association tests were conducted between the genotyped markers and the phenotype (Body weight). Two markers D5Rat256 and D5Wox37 showed a significant association with excess bodyweight as shown by the ‘F’ ratios (Table 2) suggesting for the presence of QTL. Genetic distance estimated between the two markers was 4.43cM (Figure 2). Interval mapping conducted in this genomic region resulted with LOD scores of 4.07 and 4.82 respectively for the two markers D5Rat256 and D5Wox37. The estimated LOD significance threshold (LOD = 1.92, for p=0.01) has confirmed the suggestive nature of the QTL. Confidence intervals (CI) generated from bootstrap analysis resulted with LOD scores 3.0 and 3.88 respectively at the two markers, D5Rat256 and D5Wox37. The proportion of phenotypic variance (R^2^) explained by the two markers was 10.35% and 27.48% respectively. Rat genome database shows 42 QTLs [32] for various disorders which overlap with the genomic region flanked by the two markers D5Rat256 and D5Wox37 and among them, many QTLs were found to be associated with metabolic syndrome [33-36]. Further, Rat Genome Database shows 61 genes between the two linked markers (Table S2). Leptin Receptor, the well known candidate gene for obesity is also present between the two linked markers. Multiple polymorphisms have been reported at Leptin receptor causing obesity phenotype in various rodent strains [7] and mutations in most of them were recessive in nature. While in others the mutation was on leptin receptor, it doesn’t seem to be so in WNIN/Ob as evidenced from the interval mapping. The most likely or the putative QTL position showed a peak LOD score of 4.87( p=0 ) and was distanced 0.76 cM upstream of D5Wox37 (Location of leptin receptor). Further the estimated effects of the putative QTL was found to be additive in nature (a±se= 44.8±38.4) and the additive effect of the phenotype was noticed towards substituting obese allele for the normal allele (Figure 3). Since, coding sequence for Leptin receptor seems to be unaltered in this mutant model, mapping of the putative QTL upstream of this gene suggests that the mutation in WNIN/Ob is novel and hitherto not reported. Mapping of the many QTLs for metabolic syndrome in different rodent models in this specific genomic region makes it a ‘hot spot’. Further analysis of this genomic region in WNIN/Ob should reveal novel mutation associated with development of obesity in this strain.

**Table 2:** Association test between marker and body weight.

Marker	F	P-value
D5Rat98	1.77	0.19
D5Got131	4.97	0.03
D5Rat256*	20.24	2.9E-05
D5Wox37*	25.00	4.4E-06
D5Rat235	0.36	0.55
D5Rat69	0.08	0.77

* Markers showed significant ‘F’ ratios for their association with body weight

## Supporting Information

Table S1 **F_2_ progeny genotype data and corresponding animal body weights (g).** A= WNIN/Ob alleles; H= WNIN/Ob alleles & Fisher -344 alleles; - missing data.
(DOCX)

Table S2 **Gene annotation - physical versus genetic distance.** Physical position of the genes was mined from Rat Genome Database. Physical position is indicated in Million bases (Mb) and represent the centre position of gene ((start position + stop position) /2). Genetic distance (cM) was obtained by fitting linear regression for Physical vs Genetic distance of five markers D5Rat98, D5Got131, D5Rat256, D5Wox37, D5Rat235. Equation obtained from trend line, y = 0.3356(x)-49.378. R^2^=0.9899.
(DOCX)
