# Supplementary material for: Obese Locus in WNIN/Obese Rat Maps on Chromosome 5 Upstream of Leptin Receptor
Source: PLoS One. 2013 Oct 21;8(10):e77679. doi: 10.1371/journal.pone.0077679 (PMC3804619; doi:10.1371/journal.pone.0077679)
Supplement: Table S1 — F2 progeny genotype data and corresponding animal body weights (g). A= WNIN/Ob alleles; H= WNIN/Ob alleles & Fisher -344 alleles; - missing data. (DOCX) [file pone.0077679.s001.docx]

**Table S1:**

F_2_ progeny genotype data and corresponding animal body weights (g)

|  | Animal No. | D5Rat98 | D5Got131 | D5Rat256 | D5WOX37 | D5Rat235 | D5Rat69 | Weight |
| --- | --- | --- | --- | --- | --- | --- | --- | --- |
| obese | 1 | A | A | A | A | H | H | 460 |
|  | 2 | A | A | A | A | A | A | 466 |
|  | 3 | A | A | A | A | A | H | 579 |
|  | 4 | A | A | A | A | A | H | 507 |
|  | 5 | A | A | A | A | A | A | 630 |
|  | 6 | A | A | A | A | A | A | 450 |
|  | 7 | A | A | A | A | H | H | 502 |
|  | 8 | H | H | A | A | A | A | 460 |
|  | 9 | H | H | A | A | A | A | 622 |
|  | 10 | A | A | - | A | H | H | 650 |
|  | 11 | H | H | A | A | A | H | 605 |
|  | 12 | A | A | A | A | A | A | 608 |
|  | 13 | H | A | A | A | A | A | 560 |
|  | 14 | H | A | A | A | A | H | 385 |
|  | 15 | A | - | A | A | A | A | 430 |
|  | 16 | H | A | A | A | A | H | 630 |
|  | 17 | H | H | H | A | A | A | 530 |
|  | 18 | A | A | A | A | A | A | 460 |
|  | 19 | A | A | A | A | A | A | 435 |
|  | 20 | A | A | A | A | A | A | 514 |
|  | 21 | A | A | A | A | A | A | 479 |
|  | 22 | H | H | A | A | A | A | 650 |
|  | 23 | A | A | A | A | A | A | 530 |
|  | 24 | A | A | A | A | - | - | 594 |
|  | 25 | A | A | A | A | A | H | 513 |
|  | 26 | A | A | A | A | H | H | 594 |
|  | 27 | A | A | A | A | A | H | 520 |
|  | 28 | A | A | A | A | H | H | 570 |
|  | 29 | A | A | A | A | H | H | 610 |
|  | 30 | A | A | - | A | A | H | 590 |
|  | 31 | A | A | A | A | A | A | 430 |
|  | 32 | A | A | A | A | A | H | 440 |
|  | 33 | A | A | A | A | A | A | 419 |
|  | 34 | A | A | A | A | A | A | 417 |
|  | 35 | H | A | A | A | A | A | 420 |
|  | 36 | A | A | A | A | A | A | 428 |
|  | 37 | H | A | A | A | A | A | 502 |
|  | 38 | A | A | A | A | A | A | 470 |
|  | 39 | A | A | A | A | A | A | 550 |
|  | 40 | A | A | A | A | A | A | 550 |
|  | 41 | A | A | A | A | A | H | 460 |
|  | 42 | A | A | A | A | A | A | 380 |
|  | 43 | A | A | A | A | A | A | 460 |
|  | 44 | A | A | A | A | A | H | 530 |
|  | 45 | H | A | A | A | A | A | 596 |
|  | 46 | H | H | H | A | A | H | 420 |
|  | 47 | A | A | A | A | A | A | 440 |
|  | 48 | A | A | A | A | A | H | 471 |
|  | 49 | H | H | H | A | A | H | 367 |
|  | 50 | H | H | H | A | A | A | 485 |
|  | 51 | A | A | A | A | A | A | 491 |
|  | 52 | A | A | A | A | H | H | 508 |
|  | 53 | A | A | A | A | H | - | 400 |
|  | 54 | A | A | A | A | A | A | 517 |
|  | 55 | - | A | A | A | A | H | 550 |
|  | 56 | H | H | H | A | A | H | 420 |
|  | 57 | A | A | A | A | A | A | 546 |
|  | 58 | A | A | A | A | A | H | 470 |
|  | 59 | H | H | A | A | A | H | 450 |
|  | 60 | H | H | H | A | A | A | 330 |
|  | 61 | - | A | A | A | A | H | 562 |
|  | 62 | A | A | A | A | A | - | 458 |
|  | 63 | A | A | A | A | A | A | 545 |
|  | 64 | A | A | A | A | A | A | 506 |
|  | 65 | A | A | A | A | A | H | 412 |
|  | 66 | A | A | A | A | A | A | 501 |
| carrier | 67 | H | H | H | H | H | H | 241 |
|  | 68 | H | H | H | H | H | - | 224 |

A= WNIN/Ob alleles; H= WNIN/Ob alleles and Fisher -344 alleles; - missing data
