# Supplementary material for: Obese Locus in WNIN/Obese Rat Maps on Chromosome 5 Upstream of Leptin Receptor
Source: PLoS One. 2013 Oct 21;8(10):e77679. doi: 10.1371/journal.pone.0077679 (PMC3804619; doi:10.1371/journal.pone.0077679)
Supplement: Table S2 — Gene annotation - physical versus genetic distance. Physical position of the genes was mined from Rat Genome Database. Physical position is indicated in Million bases (Mb) and represent the centre position of gene ((start position + stop position) /2). Genetic distance (cM) was obtained by fitting linear regression for Physical vs Genetic distance of five markers D5Rat98, D5Got131, D5Rat256, D5Wox37, D5Rat235. Equation obtained from trend line, y = 0.3356(x)-49.378. R2=0.9899. (DOCX) [file pone.0077679.s002.docx]

**Table S2: Gene annotation - physical versus genetic distance.**

Physical position of the genes was mined from Rat Genome Database. Physical position is indicated in Million bases (Mb) and represent the centre position of gene ((start position + stop position) /2). Genetic distance (cM) was obtained by fitting linear regression for Physical vs Genetic distance of five markers D5Rat98, D5Got131, D5Rat256, D5Wox37, D5Rat235. Equation obtained from trend line, y = 0.3356(x)-49.378. R^2^=0.9899.

| mb | cM | *Gene* |
| --- | --- | --- |
| 171.54 | 8.19 | **D5Rat256** |
| 171.99 | 8.34 | *hypothetical LOC100363381* |
| 172.23 | 8.42 | *caspase activity and apoptosis inhibitor 1* |
| 172.32 | 8.45 | *phospholipase A2, activating protein* |
| 172.42 | 8.48 | *intraflagellar transport 74 homolog (Chlamydomonas)* |
| 172.41 | 8.48 | *leucine rich repeat containing 19* |
| 172.65 | 8.56 | *TEK tyrosine kinase, endothelial* |
| 172.83 | 8.62 | *equatorin, sperm acrosome associated* |
| 172.89 | 8.64 | *myb-like, SWIRM and MPN domains 1* |
| 173.04 | 8.69 | *hypothetical protein LOC100363437* |
| 173.04 | 8.69 | *jun proto-oncogene* |
| 174.02 | 9.02 | *FGGY carbohydrate kinase domain containing* |
| 174.52 | 9.19 | *hook homolog 1 (Drosophila)* |
| 174.65 | 9.24 | *cytochrome P450, family 2, subfamily j, polypeptide 13* |
| 174.73 | 9.26 | *cytochrome P450, family 2, subfamily j, polypeptide 10* |
| 174.81 | 9.29 | *similar to cytochrome P450, family 2, subfamily j, polypeptide 11* |
| 174.94 | 9.33 | *cytochrome P450, family 2, subfamily j, polypeptide 16* |
| 175.07 | 9.37 | *cytochrome P450, family 2, subfamily j, polypeptide 4* |
| 175.13 | 9.39 | *cytochrome P450, family 2, subfamily j, polypeptide 3* |
| 175.15 | 9.40 | *similar to Ab2-162* |
| 175.16 | 9.41 | *cytochrome P450, family 2, subfamily j, polypeptide 5, pseudogene* |
| 175.33 | 9.46 | *similar to hypothetical protein MGC34837* |
| 175.45 | 9.50 | *similar to BWK-1* |
| 175.46 | 9.51 | *similar to TBC1 domain family, member 5* |
| 175.62 | 9.56 | *similar to glyceraldehyde-3-phosphate dehydrogenase* |
| 176.89 | 9.99 | *similar to 60S acidic ribosomal protein P1* |
| 177.41 | 10.16 | *nuclear factor I/A* |
| 177.85 | 10.31 | *similar to ribosomal protein L21* |
| 178.11 | 10.40 | *ribosomal protein S18-like* |
| 178.17 | 10.42 | *TM2 domain containing 1* |
| 178.20 | 10.43 | *hypothetical protein LOC685326* |
| 178.37 | 10.48 | *InaD-like (Drosophila)* |
| 178.72 | 10.60 | *LINE-1 type transposase domain containing 1* |
| 178.74 | 10.61 | *Rho GDP dissociation inhibitor (GDI) alpha-like* |
| 178.79 | 10.62 | *KN motif and ankyrin repeat domains 4* |
| 179.00 | 10.70 | *similar to ubiquitin specific protease 15* |
| 179.05 | 10.71 | *ubiquitin specific peptidase 1* |
| 179.15 | 10.74 | *dedicator of cytokinesis 7* |
| 179.22 | 10.77 | *angiopoietin-like 3* |
| 179.79 | 10.96 | *autophagy related 4C, cysteine peptidase* |
| 179.87 | 10.99 | *hypothetical protein LOC685453* |
| 179.95 | 11.01 | *similar to hypothetical protein DKFZp434P0316* |
| 180.12 | 11.07 | *similar to glyceraldehyde-3-phosphate dehydrogenase* |
| 180.27 | 11.12 | *hypothetical LOC100362448* |
| 180.38 | 11.16 | *similar to ribosomal protein L21* |
| 180.38 | 11.16 | *similar to non-SMC element 1 homolog* |
| 180.50 | 11.20 | *forkhead box D3* |
| 180.63 | 11.24 | *ALG6, alpha-1,3-glucosyltransferase* |
| 180.72 | 11.27 | *integrin beta 3 binding protein (beta3-endonexin)* |
| 180.81 | 11.30 | *EF-hand calcium binding domain 7* |
| 180.92 | 11.34 | *phosphoglucomutase 1* |
| 181.30 | 11.46 | *receptor tyrosine kinase-like orphan receptor 1* |
| 181.73 | 11.61 | *similar to hypothetical protein MGC35130* |
| 182.08 | 11.73 | *40S ribosomal protein SA-like* |
| 182.26 | 11.79 | *cache domain containing 1* |
| 182.51 | 11.87 | *hypothetical protein LOC100359934* |
| 182.63 | 11.91 | *ribonucleoprotein, PTB-binding 2* |
| 182.76 | 11.96 | *Janus kinase 1* |
| 183.12 | 12.08 | *adenylate kinase 4* |
| 183.31 | 12.14 | *DnaJ (Hsp40) homolog, subfamily C, member 6* |
| 183.48 | 12.20 | *leptin receptor overlapping transcript* |
| 183.57 | 12.23 | *leptin receptor* |
| 183.58 | 12.23 | **D5Wox37** |
